# Physicochemical and microbiological characterization of the sensory deviation responsible for the origin of the special sherry wines "palo cortado" type

**DOI:** 10.1371/journal.pone.0208330

**Published:** 2018-12-12

**Authors:** Victor Palacios, Ana Roldán, Ana Jiménez-Cantizano, Antonio Amores-Arrocha

**Affiliations:** 1 Department of Chemical Engineering and Food Technology, Faculty of Sciences, University of Cadiz, Cadiz, Spain; 2 Agrifood Campus of International Excellence (ceiA3), IVAGRO, Puerto Real, Cadiz, Spain; Maharshi Dayanand University, INDIA

## Abstract

The aim of this study was to characterize the biochemical changes and microbiological processes involved in the sensory deviation of “sobretablas” wines during biological aging, which leads to the origin of special or rare “palo cortado” wines. Industrial trials of biological aging of “sobretablas” wines with the potential for the development of lactic acid bacteria (LAB) were performed to study this phenomenon. The results showed that sensory deviation was due to the development of malolactic fermentation (MLF) together with an attenuated aerobic metabolism of “flor” yeast. Malolactic fermentation (MLF) was promoted by the presence of malic acid concentrations that were higher than 1 g/L and the coexistence of LAB and “flor” velum yeast. Ethyl lactate, acetoin and 2,3-butanediol are some of the volatile compounds that are responsible for this sensory deviation. Wines with high levels of gluconic and malic acids (> 1 g/L) can cause, with very low probability, the sensory deviation of “palo cortado”. A lysozyme dose of 12 g/hL is an effective treatment to avoid malolactic fermentation (MFL) and sensory deviation. Understanding the biochemical and microbiological changes involved in sensory deviation can be useful to wineries as markers to identify the origin of the special sherry wines "palo cortado" type.

## Introduction

Currently, the main wines produced in the Jerez region (“fino”, “manzanilla”, “oloroso” and “amontillado” sherry types) have very well-defined processes and phenomena that characterize their sensory properties. On the one hand, “fino” and “manzanilla” wines are obtained through a long process of biological aging during which they are affected by the action of a biofilm of *Saccharomyces* yeast types, called “flor” velum yeast, which develops spontaneously after alcoholic fermentation and fortification of wines at 15–15.5% alcohol content [1–5]. On the other hand, the “oloroso” sherry type is obtained after a long oxidative aging process after the fermentation and fortification of wines to 17–18% alcohol to stop the growth of "flor" yeast [1, 6]. Finally, the “amontillado” sherry type is obtained by means of biological aging, as a "fino" wine first and oxidative aging second, as in "oloroso" wines [1, 4, 7]. Both biological and oxidative aging are carried out in American oak casks. There is extensive knowledge regarding the biochemical, microbiological and physicochemical processes that are involved in both biological and oxidative aging [8–11]. The nature and chemical composition of the primary wines produced as the "fino", "oloroso" and "amontillado" types have been well-studied [12, 13, 14, 7].

However, there are currently other types of sherry wines, such as "palo cortado", which have not been characterized from an analytical point of view, and there is no literature about the biochemical or microbiological processes and phenomena involved in their production. Many authors indicate that “palo cortado”, in its most recent history, is a wine that arises spontaneously during the initial phase of biological aging of the “sobretablas” type or fortified young wines at 15–15.5% v/v [15]. The “sobretablas” are wines that have an initial stage of biological aging in casks in a static vintage sherry system for a period between 6 months and 2 years before entering the traditional "criaderas and solera” dynamic system [1]. Traditionally, there have been very few wineries that make the wine in this way, and at the end of this aging period, the "sobretablas" were classified cask-by-cask from the sensorial point of view, generally in two categories or types. On the one hand, the wines that had acquired great finesse, pallor and a pronounced pungent character continued the biological aging process and passed into the "criaderas and solera" system of "fino" wines. On the other hand, the wines that had presented a nose profile of clean and healthy wines together with a greater sensation of corpulence in the mouth, were assigned to "palo cortado" wines [15]. The "palo cortado" wines were alcoholized at 17–17.5% v/v to finish the biological aging process and to initiate oxidative aging in the vintage sherry system (usually lasting one to three years) before entering the "criaderas and solera" system, where the wine was aged for a long time until bottling [15–17].

Traditionally, the “sobretablas” wine classification was carried out by very experienced foremen/winemakers from the wineries who transmitted their selection criteria from one generation to the next. The sensory deviation, which gave rise to the "palo cortado" sherry wine type, was accidentally produced without establishing in principle any guidelines, operations or processes to condition them to occur. Unfortunately, this practice is disappearing, especially in wineries where the initial phase of "sobretablas" was carried out in stainless steel tanks, without biological aging and with very stable wines from the microbiological point of view. As a result of this lack of definition in the process, the production of "palo cortado" has always been wrapped in a halo of mystery, which has aroused the curiosity and controversy of critics and specialists in the wine world.

If sensory deviation occurs in the “sobretablas” wine during its initial phase of biological aging, it can be hypothesized that this deviation may have a biological origin that is related to “flor” yeast, 95% of which consists of strains of *Saccharomyces cerevisiae* [2, 5, 18]. In addition, this phenomenon could be due to other microorganisms that coexist with the flor yeast velum, such as lactic acid bacteria (LAB), acetic acid bacteria (AAB) or *non-Saccharomyces* yeast [19–21].

A possible sensory deviation that is characterized during biological aging is attributed to the predominant breed in the flower veil, *Saccharomyces beticus* or *Saccharomyces montuliensis* [22]. While *Saccharomyces beticus* produces wines with a more complex profile and less pungent character, *Saccharomyces montuliensis* produces high levels of acetaldehyde-marked wines. However, this is not considered a sensory deviation but a characteristic style within the fino wine typology.

Another well-characterized sensory deviation in biological aging is the increase in lactic acid levels and volatile acidity that occurs in wines due to the consumption of high gluconic acid content (> 600 mg/L) by LAB [23], which, in some cases, can lead to the "ropy" phenomenon [20, 24]. However this sensory deviation could be avoided using non-*Saccharomyces*. Some authors show that *Schizosaccharomyces pombe* can be effectively used in winemaking processes to remove gluconic acid from must prior to alcoholic fermentation [25–27]. Likewise, some strains of these yeasts can be used to reduce malic acid levels and total acidity during alcoholic fermentation [28], minimizing the development of BAL. In order to avoid a drastic reduction of the acidity, especially in warm climate wines, some authors propose the combined use of *Schizosaccharomyces pombe* and *Lachancea thermotolerans*, due to their capacity to produce lactic acid minimizing the loss of acidity [29].The sensory deviation produced by the development of AAB is also well defined and characterized, generally due to the downfall of the velum by high temperatures and/or volatile acidity that are often caused by the bacterial processes or *Brettanomyces* yeast [19, 30, 31]. The development of AAB produces significant increases in volatile acidity that affect the sensory profile of wine [32]. When the velum sinks due to high temperatures, which cause the dissolution of oxygen in the wine and the consequent oxidation of polyphenols and volatile compounds that are generated during aging, a sensory deviation characteristic of oxidation is produced that is irreversible at times and leads to wines being alcoholized up to 17.5–18% v/v to start an oxidative aging [1].

The main objective of this study is to characterize the nature of the sensory deviation that gives rise to the classification of "palo cortado" wines. In this study investigates the physicochemical and microbiological processes that are involved in this deviation. The main physicochemical markers of “flor” yeast and LAB metabolism (alcohol degree, volatile acidity, pH, malic acid, citric acid, lactic acid, gluconic acid, glycerin, acetaldehyde, acetoin, 2,3-butanediol, ethyl lactate and ethyl acetate) and bacterial populations (LAB and AAB) were analyzed. For this purpose, industrial trials of biological aging of “sobretablas” wines were performed to study the phenomena origin and processes that are involved in the sensory deviation of the “palo cortado” sherry type of wine.

## Materials and methods

### Industrial experimental design

#### “*Sobretablas*” wines selection

The “sobretablas” wine is a young wine of the *Palomino fino* variety that is fortified with ethanol (from wine distillation) to a 15% v/v alcohol content after fermentation is finished. The “sobretablas” wines undergo a biological aging process in a vintage system, which can last between 6 months and 2 years. To analyse and study the origin of “palo cortado” wines, we investigated the development of three sets of industrial “sobretablas” obtained from the winery of the “Cooperativa Andaluza, Unión de Viticultores Chiclaneros”, analytically selected based on their potential for the development of lactic bacteria during the biological aging process [23]. Three “sobretablas” (S/T) from the 2014 harvest, from three different Jerez vineyard zones, were selected according to their L-malic and gluconic acid contents, were alcoholized up to 15% v/v and were stored in stainless steel tanks in volumes of approximately 70,000 litres. The S/T_A_ had low malic acid content (< 0.5 g / L) and gluconic acid (< 0.5 g/L), and is therefore representative of “sobretablas” wines with a lower potential for lactic bacteria development. S/T_B_ had a high malic acid content (> 1 g/L) and a low gluconic acid content (< 0.5 g/L), and it is representative of “sobretablas” wines with potential for malolactic fermentation (MLF). Finally, the S/T_C_ had a high malic acid (> 1 g/L) and gluconic acid content (> 1 g/L), and it is representative of “sobretablas” wines with potential for development of malolactic and heterolactic fermentation [23, 33]. The S/T_C_ was made from harvested grapes that were affected by *Botrytis cinerea*.

#### Velum yeast

For “flor” velum yeast development, which is a strain recovered from the biological aging system of a winemaking company in the Sherry region and identified as *Saccharomyces cerevisiae*, was used (strain codified as ‘‘B16”) [2]. A filmogen culture that was used in biological aging was used as a starter of the yeast strain for inoculation. The starter cultures were prepared in an open stainless steel tank of 500 litre capacity using “sobretablas” wine that was previously filtered with a 0.45-mm membrane filter.

#### Lysozyme

The lysozyme (hydrochloride form) that was used was Enovinlyso (Agrovin, S.A., Ciudad Real, Spain). According to commercial instructions, this lysozyme was extracted from a hen egg and had a protein purity of 100% and an FIP activity > 35,000 IU/mg. An FIP unit is the measurement of enzyme activity according to the test methods of the Federation Internationale Pharmaceutique. It was used at dose of 12 g/hL, indicated for the effective treatment of advances heterolactic fermentation [33].

#### Industrial assays

Each of the “sobretablas” wines (S/T_A_, S/T_B_ and S/T_C_) was distributed in approximately 40 wine barrels, each with a 600-litre capacity, which were filled to approximately 500 litres to leave a head space on the surface of the wine for “flor yeast velum” development. Prior to filling, the casks were rinsed with pressurized water and drained for 2 hours. Once the casks were filled and marked, the industrial velum yeast from the starter tank was inoculated on the surface at the rate of one cultivar spatula (approximately 200 mL) per barrel. Then, 10 casks of each of the 40 “sobretablas” casks were treated with the lysozyme at a dose of 12.5 g/hL based on the effective treatment of advanced heterolactic fermentation [33]. All barrels developed a velum that covered the entire surface in a state of "rough" aggregation after 2 months.

The “sobretablas” wine casks that were studied were allowed to biologically age for one year, after which a sensorial classification of the wines was made. During this period, the average temperature of the winery was 20°C, with minimum and maximum recorded temperatures of 15 and 25°C, respectively, and the relative humidity was maintained in a variable range between 60 and 70%.

#### “*Sobretablas*” wine classification

First, a classification was made for each series of “sobretablas” wines (S/T_A_, S/T_B_ and S/T_C_), which each filled 40 casks. The classification was made by the winemaker and the supervisor that were responsible for the winery, who had extensive experience in the classification of “palo cortado” wines.

In addition to the winery criteria, the “sobretablas” wines were classified according to the following: a) their biological aging sensory profile (BA) (perception of acetaldehyde and sharpness in the olfactory phase, very light and thin palate, with slightly bitter sensations in the gustatory phase); b) wines with a sensory deviation typified as “palo cortado” (PC) (olfactory phase very clean without volatile acidity, notes of spread milk, caramel, milk, wood and perception of volume and heaviness, lack of fineness, buttery sensation, in the taste phase); and c) wines with a clear perception of volatile acidity (VA) (acetic acid and ethyl acetate) from lactic and/or acetic bacterial spoilage. For the wines classification, samples were extracted directly from the casks using a “venencia” sampler, and after the tasting, each cask was marked with chalk, according to the established classification, BA, PC and VA. Once the casks were classified, joint samples of each of the established categories were prepared with a final volume of approximately 5 L. A sample (n = 1, V = 0.75 L) of each of the sample sets in each series of “sobretablas” were compared by a tasting panel of 20 expert sherry tasters. Sensory verification was performed for the “sobretablas” wines series that were assigned more than one sensory category. The use of the UNE EN ISO 5495 standard paired comparison test was used for the classification of “sobretablas” wines that showed two sensory categories, and the UNE EN ISO 4120 triangular test was used for the series that showed three.

#### Physicochemical and microbiological analysis

For physicochemical and microbiological characterization analysis, three samples from each “sobretablas” wine set (n = 3, V = 1 L) were taken for further analysis of the main physicochemical markers of “flor” yeast and LAB metabolism (alcohol degree, volatile acidity, pH, malic acid, citric acid, lactic acid, gluconic acid, glycerin, acetaldehyde, acetoin, 2,3-butanediol, ethyl lactate and ethyl acetate) and bacterial populations (LAB and AAB).

### Analytical measurements

Alcohol content was determined by distillation, and the measurement of the density of the distillate was made according to the official methods for the analysis of wines [34] using an electronic densimeter DMA 48 (ANTON PAAR, Net IterLab Salt, Madrid, Spain). Volatile acidity was also measured using the official methods for the analysis of wines [34]. The gluconic acid and glycerin levels were determined using an enzymatic test (R-Biopharm, Germany). Volatile compounds, such as acetaldehyde, acetoin, 2,3-butanediol, ethyl lactate and ethyl acetate contents, were determined using a gas chromatograph equipped with an FID detector (HP 5890 Series II) and a Carbowax 20 M column (50 m, 0.25 mm ID, 0.25 mm). The injector and detector temperatures were 175°C and 225°C, respectively, and the carrier gas was hydrogen. The oven temperature was maintained at 35°C for the first 5 min and then was increased to 100°C at 5°C/min. The injected sample volume was 20 μL. For the quantification and identification of volatile compounds, 4-methyl-2-pentanol was added as an internal standard, and pure standard compounds (Sigma–Aldrich Química, S.A., Madrid, Spain) were used to determine the retention times and calibration curves.

Organic acids (citric acid, malic acid and lactic acid) in samples were analysed using an ion chromatography (Metrohm 930 Compact IC Flex) with a conductivity detector. Column: Metrosep Organic Acids—250/7.8. Analysis conditions: 0.4 mmol/L, sulfuric acid + 12% acetone; sample volume 20 microliter, flow 0.4 mL/min.

For LAB counting, dilutions were prepared in a phosphate buffer (pH 6.2). After 10-fold serial dilutions were prepared, 0.1 mL of each dilution was spread onto an MRS agar plate (Sigma–Aldrich Química, S.A., Madrid, Spain). The plates were incubated at 30°C in the presence of CO_2_ (5%) for 72 h. Samples were taken daily in triplicate. For AAB counting, the same buffer and serial dilutions were used but each dilution was spread onto a GYC culture medium (5% D-glucose, 1% yeast extract, 0.5% CaCO_3_ and 2% agar) (Sigma–Aldrich Química, S.A., Madrid, Spain) [35] that was supplemented with pimaricin (50 mg/L) (Sigma–Aldrich Química, S.A., Madrid, Spain) and penicillin (12.5 mg/L) (Sigma–Aldrich Química, S.A., Madrid, Spain). The plates were incubated for 2–4 days at 28°C under aerobic conditions. The viability of the LAB and AAB was determined and expressed as colony forming units per millilitre (CFU/mL).

### Statistical analysis

Data analysis included the calculation of the means and standard deviations using the statistical package GraphPad Prism version 6.01 (GraphPad Software, San Diego, CA). Significant differences were evaluated by two-way ANOVA and Bonferroni´s multiple range (BSD) tests with a confidence level of 95% (p < 0.05).

## Results and discussion

### Assay 1. Evolution of S/T_A_ wine with malic acid low levels

First, the S/T_A_ wine with low malic acid content, low potential for LAB growth and a possibility to give “palo cortado” sensory deviation after 1 year of biological aging was studied. Table 1 shows the results of the sensory classification of the 40 casks of S/T_A_ by the winery. All tasted samples were classified as biological aging sensory profile (BA). Any of the S/T_A_ studied, including the 10 casks that were treated with lysozyme, showed sensory deviation “palo cortado” (PC) or perception of volatile acidity (VA).

**Table 1:** Sensory classification results of the "sobretablas" (S/T) made by the winery after 1 year of biological aging.

N = 40 casks	Palo Cortado (PC)	Biological Aging (BA)	Volatile Acidity (VA)
S/T_A_	0	30 + 10*	0
S/T_B_	12	18 + 10*	0
S/T_C_	3	15 + 10*	12

N = 40 casks for each type of "sobretablas". Categories of classification: Palo Cortado (PC), Biological Aging (BA) and Volatile Acidity (VA).

* Casks treated with lysozymes 12.5 g/hL

As shown in Table 2, the wine sets S/T_A_ (BA) and S/T_A_ lys (BA) show the characteristic evolution of biologically aged wines, with respect to the control wine at t = 0 (S/T_A_ t = 0). During this process, “flor” velum yeast consumes ethanol, glycerine and acetic acid (volatile acidity), and produce acetaldehyde, acetoin and 2,3-butanediol [10, 36, 11]. Likewise, it can be observed that there is a low reduction of malic acid in the S/T_A_ (BA) set, which could be attributed to a certain consumption by the small population of LAB that was detected (570 CFU/mL) (Table 2). It is very common that during the biological aging in the presence of “flor” velum, a certain population of LAB coexists [21, 20] due to the semi-anaerobic conditions that are established by the aerobic metabolism of “flor” velum yeast. Anyway, the development of lactic acid bacteria and the MFL process is attenuated, which could be mainly due to the initial low malic acid concentrations. This finding could be corroborated when LAB is not found due to lysozyme treatment (S/T_A_ lys (BA)) (Table 2). Consequently, the malic acid levels remain unchanged or similar to those of the control.

**Table 2:** Evolution of analytical composition of the sets of "sobretablas" casks S/T_A_ classified as BA after 1 year of aging.

	S/T_A_ t = 0	S/T_A_ (BA)	S/T_A_ lys (BA)
Alcohol degree (% v/v)	15.1^a^ ± 0.1	14.8^a^ ± 0.1	14.9^a^ ± 0.1
Volatile acidity (g/L)	0.25^a^ ± 0.01	0.19^a^ ± 0.02	0.18^a^ ± 0.01
pH	3.27^a^ ± 0.09	3.21^a^ ± 0.07	3.23^a^ ± 0.03
Malic acid (g/L)	0.54^a^ ± 0.02	0.45^b^ ± 0.01	0.53^a^ ± 0.02
Citric acid (g/L)	0.39^a^ ± 0.06	0.25^b^ ± 0.02	0.35^a^^,^^b^ ± 0.03
Lactic acid (g/L)	0.25^a^ ± 0.03	0.19^a^^,^^b^ ± 0.02	0.10^b^ ± 0.01
Gluconic acid (g/L)	0.48^a^ ± 0.02	0.37^b^ ± 0.03	0.61^c^ ± 0.02
Glycerine (g/L)	6.8^a^ ± 0.3	3.4^b^ ± 0.5	3.1^c^ ± 0.1
Acetaldehyde (mg/L)	54^a^ ± 2	142^b^ ± 6	132^b^ ± 3
Acetoin (mg/L)	0.89^a^ ± 0.03	7.39^b^ ± 0.9	8.69^b^ ± 0.5
2,3-butanediol (mg/L)	69^a^ ± 1	530^b^ ± 18	409^c^ ± 11
Ethyl lactate (mg/L)	34.2^a^ ± 2.4	92.4^b^ ± 1.6	58.6^c^ ± 1.9
Ethyl acetate (mg/L)	45.6^a^ ± 1.1	52.7^b^ ± 1.3	41.2^c^ ± 1.1
LAB (CFU/mL)	121^a^ ± 29	570^b^ ± 68	n.f.
AAB (CFU/mL)	n.f.	n.f.	n.f.

S/T_A_ t = 0 or control: initial “sobretablas” wine at t = 0; BA: biological aging; S/T_A_ lys (BA): set of "sobretablas" classified as BA that has been treated initially with 12 g/hL of lysozyme.

^a,b,c^ Different lowercase superscript letters mean statistically significant differences between samples at p < 0.05 obtained by two-way ANOVA and Bonferroni´s multiple range (BSD) test. Results are the means ± SD of three repetitions. n.f.: not found.

Malic acid consumption by LAB in S/T_A_ (BA) does not correspond to an increase in lactic acid due to consumption by the “flor” velum yeast [8]. Lactic acid levels show a reduction of 24% in S/T_A_ (BA) and 60% in S/T_A_ lys (BA), both with respect to the control. This major decrease in S/TA lys (BA) could be attributed to that there is no MLF. As a result, ethyl acetate, ethyl lactate and 2,3-butanediol levels in S/TA lys (BA) are significantly lower than in S/TA (BA) (Table 2).

### Assay 2. Evolution of S/T_B_ wine with high malic acid levels

In the second assay, the goal is to analyse whether a S/T_B_ wine with LAB growth potential could produce a “palo cortado” sensory deviation over the course of one year of biological aging.

The sensory classification of the S/T_B_ casks that were made by the winery established two distinct groups (Table 1). Twelve casks were classified as “palo cortado” (PC), and the other casks were classified as biological aging (BA) wines, including the 10 casks that were treated with lysozyme. All wines classified as PC (40%) were derived exclusively from S/T_B_ wine not treated with lysozyme, and no samples presented a sensory deviation of type VA.

To verify significant differences between the two sets of wines PC and BA, a sensory analysis was made in pairs according to UNE EN ISO 5495 (Table 3). The results obtained from the paired comparison test showed that samples (PC and BA) were significantly different in the panel test, and 85% of the panelists were able to detect a difference between groups PC and BA with an α_risk_ = 0.01.

**Table 3:** Verification test of the sensory classification process of palo cortado (PC), biological aging (BA) and volatile acidity (VA) set of wines by tasting panel (N = 20).

N = 20 panelists	(a) Palo Cortado (PC)–Biological Aging (BA)	(b) Palo Cortado (PC)–Volatile Acidity (VA)	(c) Biological Aging (BA)–Volatile Acidity (VA)
S/T_B_ *	17 ^a^	-	-
S/T_C_**	15 ^b^	13 ^a^	18 ^b^

N = number of panelists that differentiate wine classes by applying UNE EN ISO standards.

^a,b^ Superscript mean confidence level (α risk) in the wines: ^a^ α_risk_ < 0,01 and ^b^ α_risk_ < 0,001

^*^ UNE EN ISO 5495

^**^ UNE EN ISO 4120

Table 4 shows the results of the main markers of biological aging and MLF together with LAB and AAB populations, analysed for the following wine classified sets: S/T_B_ (PC), S/T_B_ (BA), and S/T_B_ lys (BA), and the control wine at t = 0 (S/T_B_ t = 0). With respect to the other samples and control, S/T_B_ (PC) had significant residual levels of acid malic (0.21 g/L) and citric acid (0.10 g/L), and very high levels of lactic acid (1.2 g/L). This could be due to an intensive MLF that leads to the conversion of malic acid into lactic acid by LAB [37–39]. High populations of LAB (35000 CFU/mL) that were detected in S/T_B_ (PC) could justify this phenomenon (Table 4).

**Table 4:** Evolution of analytical composition of the S/T_B_ "sobretablas" casks sets classified as PC and BA after 1 year of aging.

	S/T_B_ t = 0	S/T_B_ (PC)	S/T_B_ (BA)	S/TB lys (BA)
Alcohol degree (% v/v)	15.2^a^ ± 0.1	15.1^a^ ± 0.1	14.8^a^ ± 0.1	15.0^a^ ± 0.1
Volatile acidity (g/L)	0.24^a^ ± 0.01	0.24^a^ ± 0.01	0.21^a^ ± 0.03	0.21^a^ ± 0.01
pH	3.22^a^ ± 0.03	3.22^a^ ± 0.01	3.18^a^ ± 0.02	3.25^a^ ± 0.03
Malic acid (g/L)	1.75^a^ ± 0.04	0.21^b^ ± 0.02	1.48^c^ ± 0.06	1.78^a^ ± 0.04
Lactic acid (g/L)	0.36^a^ ± 0.01	1.21^b^ ± 0.06	0.35^a^ ± 0.01	0.21^c^ ± 0.01
Citric acid (g/L)	0.42^a^ ± 0.03	0.10^b^ ± 0.04	0.39^a^ ± 0.04	0.37^a^ ± 0.02
Gluconic acid (g/L)	0.44^a^ ± 0.02	0.35^a^ ± 0.02	0.39^a^ ± 0.06	0.42^a^ ± 0.02
Glycerine (g/L)	7.5^a^ ± 0.1	6.5^b^ ± 0.1	5.3^c^ ± 0.2	5.1^d^ ± 0.2
Acetaldehyde (mg/L)	52^a^ ± 3	71^a^ ± 9	102^a^ ± 4	129^a^ ± 13
Acetoin (mg/L)	0.9^a^ ± 0.1	62.6^b^ ± 1.5	35.2^c^ ± 1.7	28.3^c^ ± 0.5
2,3-butanediol (mg/L)	47^a^ ± 2	850^c^ ± 11	670^b^ ± 11	620^b^ ± 6
Ethyl lactate (mg/L)	29.8^a^ ± 1.3	329^b^ ± 3.2	77.1^a^ ± 3.2	42.8 ^a^ ± 0.9
Ethyl acetate (mg/L)	32.7^a^ ± 2.3	65.2^a^ ± 1.8	52.7^a^ ± 1.8	49.2^a^ ± 0.9
LAB (CFU/mL)	171^a^ ± 7	35123^b^ ± 102	765^c^ ± 35	2^d^ ± 1
AAB (CFU/mL)	n.f.	n.f.	n.f.	n.f.

S/T_B_ t = 0 or control: initial “sobretablas” wine at t = 0; BA: biological aging; PC: “Palo cortado”; S/T_B_ lys BA initially treated with 12 g/hL of lysozyme.

^a,b,c,d^ Different lowercase superscript letters mean statistically significant differences between samples at p < 0.05 obtained by two-way ANOVA and Bonferroni´s multiple range (BSD) test. Results are the means ± SD of three repetitions. n.f.: not found.

Acetoin and 2,3-butanediol levels in the S/T_B_ (PC) are significantly higher (62,6 mg/L and 850 mg/L, respectively) than those in the other categories. Acetoin and 2,3-butanediol levels in S/T_B_ (PC) are approximately 77% and 26%, both higher compared to S/T_B_ (BA) wines. A large number of studies have found that acetoin and 2-3-butanediol can be produced by LAB, mainly through the metabolism of citric acid during MLF [40, 41]. In addition, during biological aging, acetoin can also be generated from acetaldehyde through reductive acetonic condensation, and its subsequent reduction can lead to 2,3-butanediol [42, 43, 8]. Acetoin and 2,3-butanediol levels that were attained in the S/T_B_ (PC) are above the thresholds of perception (30 mg/L and 668 mg/L respectively), both with lactic aromas, butter, cream, etc. [44, 6], and therefore could contribute to the sensory deviation that is characteristic of these wine types.

S/T_B_ (PC) have low levels of volatile acidity, approximately 0.24 g/L, which is very similar to those with S/T_B_ (BA), and S/T_B_ lys (BA). This result indicates that the “flor” velum yeast assimilates the small proportion of acetic acid produced by LAB during MLF.

As expected, the levels of ethyl lactate that are achieved in the S/T_B_ (PC) are significantly higher than those of S/T_B_ (BA) and S/T_B_ lys (BA), possibly due to LAB esterases action. Several studies have confirmed that LAB esterases are responsible for ethyl lactate production during MFL [45, 46]. Ethyl lactate levels achieved in the S/T_B_ (PC) (329 mg/L) are almost 11 times higher than the control values, much higher than the threshold perception (150 mg/L), which contributes to butterscotch and buttery odour descriptors [14].

Regarding the metabolites of biological aging, we can see in Table 4 that the levels of glycerin in S/T_B_ (PC) are significantly higher than those in S/T_B_ (BA) and S/T_B_ lys (BA). Glycerin decreases only 13% over one year of biological aging, which corresponds to a very low consumption rate when compared with S/T_B_ (BA) and S/T_B_ lys (BA), which show decreases of 42% and 50%, respectively. It is likely that the relative high glycerin levels in S/T_B_ (PC) could partially justify the perception of greater volume in the mouth that provides as a differential attribute for the identification of “palo cortado”.

Acetaldehyde showed the same behaviour, whose production by “flor” velum yeast is very low in S/T_B_ (PC) (71 mg/L) compared to that of S/T_B_ (BA) and S/T_B_ lys (BA), which present 102 mg/L and 129 mg/L, respectively. This clearly indicates that the development of “flor” velum yeast and/or their metabolism could be affected by LAB growth and MLF. In this sense, some authors have confirmed that “flor” velum yeast has growth difficulties in the film-forming phase when there is LAB growth in the medium [47]. Comparing the results obtained for S/T_B_ and S/T_A_ (Tables 2 and 4), it is clear that one of the main reasons for the appearance of the “palo cortado” profile or the "accident" is the presence of high concentrations of malic acid. Malic acid levels in S/T_B_ t = 0 (1.75 g/L) are an exceptional case for a *Palomino fino* “sobretablas” wine, which only occurs in certain vintages with a lack of maturation and comes from specific cultivation zones [48].

When we analysed S/T_B_ (BA) (Table 4), we again observed the same behaviour as in S/T_A_ (BA) (Table 2) with respect to malic, citric and lactic acid due to low LAB populations (765 CFU/mL).

It is probable that some of these “sobretablas” wines could develop an MLF over time, which is the sensory deviation characteristic of the “palo cortado”, if they continued the process of biological aging. However, when these wines are traded to a “criaderas and solera” system of “fino” wine type, they are introduced into the casks of the youngest “criaderas” at a maximum of 25% of the volume, which dilutes the malic acid and citric acid content and reduces their potential to become “palo cortado”.

Finally, in S/T_B_ lys (BA), where LAB is not detected and the MFL is not developed, initial malic and citric acid levels are maintained with respect to the control. Therefore, adding a lysozyme could be a good treatment to avoid the production of “palo cortado” wines.

### Assay 3. Evolution of S/T_C_ wine with high levels of malic and gluconic acids

In this final assay, the goal was to analyse whether a S/T_C_ wine with MLF and heterolactic fermentation potential could give rise to a “palo cortado” sensory deviation over the course of one year of biological aging.

As shown in Table 1, the sensory classification of the 40 casks of S/T_C_ wines gave rise to three differentiated groups of samples. In this case, 3 casks were classified as “palo cortado” (PC) wines, 12 casks presented high intensity in volatile acidity (VA), and all the remaining wines (25 casks), including 10 casks that had been treated with lysozymes, presented a biological aging (BA) profile. To verify significant differences between the three wine groups, a sensory analysis was performed using the triangular test according to the UNI EN ISO 4120 standard with 20 panelists. The results obtained from the triangular test (Table 3) showed that the three wines samples that were compared are significantly different, and 90% of the panelists differentiated the categories BA and VA with α risk <0.001 confidence level; a lower percentage (75%) of panelists (α_risk_ <0.001) differentiated between PC and BA; and finally, with the lowest success rate, 65% of panelists correctly differentiated between a PC and VA with α_risk_ <0.01.

Table 5 presents the analytical and microbiological results that correspond to the wine sets classified as S/T_C_ (BA), S/T_C_ (PC), S/T_C_ (VA) and S/T_C_ lys (BA), together with the control wine at t = 0 (S/T_C_ t = 0).

**Table 5:** Evolution of analytical composition of the S/T_C_ "sobretablas" casks sets classified as PC, BA and VA after 1 year of aging.

	S/T_C_ t = 0	S/T_C_ (BA)	S/T_C_ (PC)	S/T_C_ (VA)	S/T_C_ lys (BA)
Alcohol degree (% v/v)	15.1^a^ ± 0.1	14.9^a^ ± 0.1	14.9^a^ ± 0.1	14.8^a^ ± 0.2	15.0^a^ ± 0.1
Volatile acidity (g/L)	0.21^a^ ± 0.02	0.32^a^ ± 0.02	0.37^a^ ± 0.02	0.81^a^ ± 0.01	0.19^a^ ± 0.01
pH	3.18^a^ ± 0.02	3.20^a^ ± 0.01	3.22^a^ ± 0.01	3.15^a^ ± 0.02	3.21^a^ ± 0.02
Malic acid (g/L)	1.25^a^ ± 0.01	0.74^b^ ± 0.05	0.15^c^ ± 0.02	0.12^c^ ± 0.03	1.01^d^ ± 0.01
Lactic acid (g/L)	0.28^a^ ± 0.02	0.19^a^ ± 0.01	1.25^b^ ± 0.05	1.75^c^ ± 0.09	0.15^a^ ± 0.01
Citric acid (g/L)	0.30^a^ ± 0.01	0.40^a^ ± 0.04	0.11^b^ ± 0.03	0.15^b^^.^^c^ ± 0.02	0.42^a^ ± 0.03
Gluconic acid (g/L)	1.10^a^ ± 0.03	0.90^b^ ± 0.05	0.75^b^^,^^c^ ± 0.01	0.24^d^ ± 0.02	1.00^a^^,^^b^ ± 0.03
Glycerine (g/L)	9.7^a^ ± 0.2	5.2^b^ ± 0.2	7.8^c^ ± 0.3	9.3^d^ ± 0.02	5.4^e^ ± 0.1
Acetaldehyde (mg/L)	48^a^ ± 5	101^a^ ± 4	64^a^ ± 5	38^a^ ± 2	121^a^ ± 5
Acetoin (mg/L)	0.7^a^ ± 0.1	21.3^a^ ± 1.7	54.2^a^ ± 1.2	39^a^ ± 4	31^a^ ± 2
2,3-butanediol (mg/L)	39^a^ ± 5	368^b^ ± 11	548^b^^,^^c^ ± 9	512^b^^,^^d^ ± 10	540^b^^,^^e^ ± 13
Ethyl lactate (mg/L)	33.4^a^ ± 2.6	89.2^a^ ± 3.2	624^b^ ± 10	740^b^^,^^c^ ± 25	59.2^a^ ± 1.3
Ethyl acetate (mg/L)	50.8^a^ ± 3.1	71.2^a^ ± 1.8	90.1^a^ ± 1.9	450^b^ ± 12	62.3^a^ ± 2.6
LAB (CFU/mL)	294^a^ ± 12	2148^b^ ± 67	45978^c^ ± 297	98636^d^ ± 397	5^e^ ± 1
AAB (CFU/mL)	6^a^ ± 3	12^a^ ± 3	221^b^ ± 18	6241^d^ ± 121	2^a^ ± 1

S/T_C_ t = 0 or control: initial “sobretablas” wine at t = 0; BA: biological aging; PC: “Palo cortado”; VA: volatile acidity; S/T_C_ lys (BA): set of "sobretablas" classified as BA initially treated with 12 g/hL of lysozyme.

^a, b, c,d,e^ Different lowercase superscript letters mean statistically significant differences between samples at p < 0.05 obtained by two-way ANOVA and Bonferroni´s multiple range (BSD) test. Results are the means ± SD of three repetitions.

First, the results highlight that a "sobretablas" with high contents of gluconic and malic acids S/T_C_ t = 0 could lead to a “palo cortado” sensory deviation. However, the frequency of “palo cortado” sensory deviation is very low (10%), although the malic acid contents were elevated.

Malic and citric acid residual values in S/T_C_ (PC) show that MLF could be responsible for the sensory deviation in these types of wines. The levels of lactic acid (1.25 g/L) are very similar to those obtained in S/T_B_ (PC) (1.21 g/L) (Table 4). However, the levels of acetoin and 2,3-butanediol are lower, possibly due to the initial citric acid content in the control (Table 5). The relatively low levels of acetaldehyde (71 mg / L) and especially high levels of glycerine (7.8 g/L) with respect to S/T_C_ (BA) (5 g/L) again indicate that the viability and/or metabolism of “flor” velum yeast have been significantly reduced. The initial glycerine values in the control are especially high compared to the S/T_A_ control (Table 2) and S/T_B_ control (Table 4) due to the metabolism of *Botrytis cinerea* in grapes. These high glycerine values could generate a higher perception of volume in the mouth characteristics of “palo cortado in S/T_C_ (PC).

As seen in Table 5, the LAB population that was reached in S/T_C_ (PC) was relatively high (45978 CFU/mL) compared S/T_C_ (BA) and was greater than the S/T_B_ (PC) value (Table 4). This could be expected because the S/T_C_ control presented a higher LAB population. However, it should be noted that after 1 year of biological aging, the S/T_C_ (PC) had relatively high values of gluconic acid (0.78 g/L). This finding may indicate, on the one hand, that there was not significant consumption of this substrate, and that LAB preferably assimilated the malic acid or the citric acid over gluconic acid during the biological aging process. A malic acid and citric acid consumption of 88% and 77%, respectively, were produced in comparation to 31% of gluconic acid consumption. (Table 5). If LAB development continued in S/T_C_ (PC), it would be likely that LAB will tend to metabolize the remaining gluconic acid, significantly raising the volatile acidity of the wines. In this way, S/T_C_ (PC) would tend to become an S/T_C_ (VA) type, and only intense biological aging could minimize the increase in volatile acidity [47]. As the results showed (Table 5), 40% of the casks of S/T_C_, not including those treated with lysozyme, are classified as S/T_C_ (VA) presenting a LAB spoilage, and beginnings of spoilage by AAB. S/T_C_ (VA) showed high values of lactic acid (1 g/L), volatile acidity (0.81 g/L) and high populations of both LAB and AAB (Table 5).

Therefore, the sensory deviation of “palo cortado” can appear in this type of "sobretablas" wine with less than 1 year of biological aging and probably requires more periodic and early sensory analysis for its detection since the spoilage risks are important. In any case, to preserve the characteristics of “palo cortado” wines, wines should be rapidly alcoholized up to 17.5–18% v/v to inhibit all biological activity, thus initiating the oxidative aging process. In order to minimize spoilage risk and maximize the “palo cortado” sensory deviation, *Saccharomyces pombe* could be used to deplete gluconic acid in grape musts [25–27].

On the other hand, 50% of the S/T_C_ were classified as BA, 10% less than the S/T_B_. The high values in acetaldehyde and the decrease in glycerine with respect to the control (Table 5) indicate that these casks have significantly accentuated the growth and metabolism of “flor” yeast. Due to the high metabolic activity of the yeast, the lactic acid does not increase, but it decreases by almost 30%.

Finally, in this assay it is corroborated that lysozymes are an effective treatment to prevent the development and spoilage of LAB in wines during biological aging [42]. As seen in Table 5, the S/T_C_ lys (BA) present very similar values of malic, citric and gluconic acid to the control, due to the lack of LAB. The residual levels of LAB found in S/T_C_ lys (BA) (5 CFU/mL) indicate that lysozymes are very effective in preventing heterolactic fermentation during biological aging. Also, it could be interesting to use chitosan as an alternative control tool for LAB populations and other microorganisms such as *Brettanomyces* [49]

## Conclusion

Malic acid concentrations higher than 1 g/L, low level of gluconic acid and LAB presence in “sobretablas” wines during biological aging could produce a sensory deviation due to an intense development of MLF. As a consequence, some volatile compounds, such us ethyl lactate, acetoin and 2,3-butanediol increase significantly until concentration above their thresholds of perception. This compounds could contribute to the sensory deviation (butter, cream, butterscotch), giving rise to “palo cortado” wines classification. The relatively high levels of glycerine and low acetaldehyde contents, due to the attenuated aerobic metabolism of “flor” yeast, also contribute to sensory deviation, reducing the pungent character in the olfactory phase and increasing the perception of volume in the mouth, respectively. Wines with high levels of gluconic and malic acids (> 1 g/L) can give rise, with very low probability, to the sensory deviation of “palo cortado”. These wines tend to develop heterolactic fermentations that lead to a significant increase in volatile acidity and a spoilage by LAB and AAB. A lysozyme dose of 12.5 g/hL is an effective treatment for avoiding MFL, the spoilage risk and sensory deviation by LAB during biological aging.

This new understanding of the biochemical and microbiological changes involved in “sobretablas” wines sensory deviation can be useful to wineries as markers to identify the origin of the special sherry wines "palo cortado" type.
